# Evaluating current automatic de-identification methods with Veteran’s health administration clinical documents

**DOI:** 10.1186/1471-2288-12-109

**Published:** 2012-07-27

**Authors:** Oscar Ferrández, Brett R South, Shuying Shen, F Jeffrey. Friedlin, Matthew H Samore, Stéphane M Meystre

**Affiliations:** 1 Department of Biomedical Informatics, University of Utah, Salt Lake City, UT, USA; 2 IDEAS Center SLCVA Healthcare System, Salt Lake City, UT, USA; 3 Medical Informatics, Regenstrief Institute, Inc, Indianapolis, IN, USA

**Keywords:** Confidentiality, patient data privacy [MeSH F04.096.544.335.240], Natural language processing [L01.224.065.580], Health insurance portability and accountability act [N03.219.521.576.343.349], De-identification, Anonymization, Electronic health records [E05.318.308.940.968.625.500], United States department of veterans affairs [I01.409.137.500.700]

## Abstract

**Background:**

The increased use and adoption of Electronic Health Records (EHR) causes a tremendous growth in digital information useful for clinicians, researchers and many other operational purposes. However, this information is rich in Protected Health Information (PHI), which severely restricts its access and possible uses. A number of investigators have developed methods for automatically de-identifying EHR documents by removing PHI, as specified in the Health Insurance Portability and Accountability Act “Safe Harbor” method.

This study focuses on the evaluation of existing automated text de-identification methods and tools, as applied to Veterans Health Administration (VHA) clinical documents, to assess which methods perform better with each category of PHI found in our clinical notes; and when new methods are needed to improve performance.

**Methods:**

We installed and evaluated five text de-identification systems “out-of-the-box” using a corpus of VHA clinical documents. The systems based on machine learning methods were trained with the 2006 i2b2 de-identification corpora and evaluated with our VHA corpus, and also evaluated with a ten-fold cross-validation experiment using our VHA corpus. We counted exact, partial, and fully contained matches with reference annotations, considering each PHI type separately, or only one unique ‘PHI’ category. Performance of the systems was assessed using recall (equivalent to sensitivity) and precision (equivalent to positive predictive value) metrics, as well as the F_2_-measure.

**Results:**

Overall, systems based on rules and pattern matching achieved better recall, and precision was always better with systems based on machine learning approaches. The highest “out-of-the-box” F_2_-measure was 67% for partial matches; the best precision and recall were 95% and 78%, respectively. Finally, the ten-fold cross validation experiment allowed for an increase of the F_2_-measure to 79% with partial matches.

**Conclusions:**

The “out-of-the-box” evaluation of text de-identification systems provided us with compelling insight about the best methods for de-identification of VHA clinical documents. The errors analysis demonstrated an important need for customization to PHI formats specific to VHA documents. This study informed the planning and development of a “best-of-breed” automatic de-identification application for VHA clinical text.

## Background

With the increased use and adoption of Electronic Health Records (EHR) systems, we have witnessed a tremendous growth in digital information useful for clinicians, researchers and many other operational purposes. However, this vastness of data is rich in Protected Health Information (PHI), which severely restricts its access and possible uses.

In the United States, the confidentiality of patient data is protected by the Health Insurance Portability and Accountability Act (HIPAA; codified as 45 CFR §160 and 164) and the Common Rule [1]. These laws typically require the informed consent of the patient and approval of the Internal Review Board (IRB) to use data for research purposes, but these requirements are sometimes extremely difficult or even impossible to fulfill (e.g., retrospective studies of large patient populations who moved, changed healthcare system, or died). Such requirements can be waived if data is de-identified. For clinical data to be considered de-identified, the HIPAA “Safe Harbor” technique requires 18 PHI identifiers to be removed; further details regarding these 18 PHI identifiers can be found in [2,3].

As EHRs are growing exponentially and a significant amount of clinical information is stored as unstructured data, manual de-identification becomes prohibitively time consuming; the construction of automated systems capable of de-identifying clinical documents could alleviate this issue and is nowadays an important challenge for the research community.

Optimal strategies to automatically de-identify clinical documents would also facilitate the availability of clinical narratives for clinical information extraction applications development, and applications that are able to produce structured information from clinical narratives are of paramount importance for clinical research [4]. Although the potential uses of information extraction from clinical text are numerous and far-reaching, most applications are rarely applied outside of the laboratories they have been developed in, mostly because of scalability and generalizability issues, which could be surpassed if large sets of de-identified narratives were made available. This represents a strong motivation to develop automated de-identification systems and encourages us to find the best methods to de-identify our documents.

When text de-identification could not be accomplished, researchers could rely on alternative methods such as HIPAA-compliant computational protected environments, or release of derived data that do not contain PHI (e.g., aggregate data statistics). However, in this case researchers outside the institution could not take advantage of the rich information hidden within the textual content of the clinical documents.

Text de-identification involves challenges related to the extraction of precise pieces of information from unstructured data, and resembles the traditional Named Entity Recognition (NER) [5] task often addressed with Natural Language Processing (NLP) algorithms and methods. As stated in [3], most automated de-identification systems target only some types of PHI, and not all 18 classes of PHI cited in HIPAA. Furthermore, the majority of systems used only one or two specific document types, making the generalization of these methods across varied document sources difficult.

We will now briefly describe the main approaches followed to tackle the de-identification problem.

### Principal methods used for automatic de-identification

Although many systems combine different approaches to de-identify specific PHI types, we present here a broad classification depending on the main technique used to obscure PHI. Specifically, we classify de-identification tools in two groups of methodologies: rule-based and machine learning-based, as proposed in an earlier publication [3].

Rule-based systems usually tackle the de-identification task with pattern matching, regular expressions and dictionary lookups [6-9]. The major drawback of this approach is the need for experienced domain experts to manually create patterns, rules and dictionaries. This implies a tedious task with limited generalizability. Although some dictionaries are quite generalizable such as lists of first names and countries, others are built specifically for the institution in which the system was developed in (e.g., list of actual names of patients, physicians or healthcare providers). In addition, the developers of rule-based systems have to be aware of all possible PHI patterns that can occur, such as unexpected date formats or non-standard abbreviations. Rule-based methods require little or no annotated training data, and can be easily and quickly modified to improve performance by adding rules, dictionary terms, or regular expressions.

### Machine learning-based systems

Recent applications tend to mostly base their predictions on supervised machine learning methods. Although such methods require a large annotated corpus for training, a resource that requires significant work in terms of human resources, supervised machine learning methods have the advantage of automatically learning how to recognize complex PHI patterns; consequently, developers require little knowledge of PHI patterns. One disadvantage of machine learning-based systems is that they may not learn PHI patterns that occur rarely in the annotated corpus. For machine learning algorithms, feature selection is the key aspect of these systems. Most of them use a variety of features ranging from lexical features (e.g., word-level features such as word case, punctuation, special and numerical characters, and the morphology of the word) to contextual features or complex features derived, for instance, from part-of-speech tagging or lexical-semantic resources [10-16].

Therefore, although machine learning de-identification methods are typically more generalizable than rule-based methods, it is sometimes difficult to know precisely why the method committed an error and additional training data is often required when these approaches are applied to a new dataset.

The scope of this research is under the umbrella of an informatics initiative funded by the Department of Veteran’s Affairs, called the Consortium for Healthcare Informatics Research (CHIR); focusing on utilizing both structured and unstructured data previously unavailable for research and operational purposes. These efforts have also focused on creating a high-performance computing environment to support data management, analytics and development environments called the Veterans’ Informatics, Information and Computing Infrastructure (VINCI). Therefore, building tools that can be used to automatically de-identify Veteran’s Health Administration (VHA) clinical documents is of paramount importance in the development of this initiative.

To be trusted, automatic de-identification has to provide “acceptable” accuracy, but what this acceptable performance should be remains a controversial question. No accepted objective answer to this question exists, and many factors influence this answer: the final purpose of the de-identified documents, the legal agreements that could be imposed to avoid re-identification, and the fact that some PHI categories are more sensitive than others. We also believe that since patient confidentiality is critical, de-identification systems should give more emphasis to sensitivity than to positive predictive value. With this consideration in mind, and to guide our efforts, we set this “acceptable” performance to a sensitivity of 95% and a positive predictive value of 80% for highly sensitive PHI (patient names and SSN), and a sensitivity of 85% and positive predictive value of 70% for other types of PHI.

Throughout this paper, we will present an evaluation of existing automated text de-identification methods and tools applied to VHA clinical documents. The main research question guiding this evaluation is: Can existing text de-identification applications have sufficient accuracy (recall ≥ 90% and precision ≥ 80%) when used “out-of-the-box” with VHA clinical text? Two follow-up questions asked in this study are: 1) What improvement in accuracy does the re-training with the VHA corpus allow when tested with VHA clinical text?; and 2) Which methods and resources allow for the best accuracy for each PHI category? This study will eventually provide useful knowledge to and inform development of a de-identification system customized for VHA clinical documents.

## Methods

We selected five clinical text de-identification tools and evaluated them to assess the performance of current text de-identification methods with VHA clinical documents. After reviewing the literature [3], we chose successful automated clinical documents de-identification systems and selected systems that tackled the de-identification task from different perspectives and approaches in order to have a global picture of the different methods used.

The focus of this study is to identify the most successful methods for de-identifying VHA clinical documents, and although we report evaluation results for all systems tested in this study, it is not our goal to make a comparison or competition between them.

### Main characteristics of selected rule-based de-identification systems

. *HMS Scrubber*[6]. HMS Scrubber is a freely available open source tool developed within the Shared Pathology Informatics Network (SPIN). It was designed to obscure HIPAA identifiers and tailored to pathology reports. As part of the SPIN project, an XML schema was defined to accommodate different information contained within a pathology report; HMS Scrubber can operate with such an XML schema as well as plain-text documents. The algorithmic structure of HMS Scrubber involves the following steps: (1) if the reports follow the SPIN XML schema, HMS Scrubber takes advantage of the predefined information and searches for any occurrences of these identifiers in the textual portion of the document; (2) pattern matching with regular expressions, 50 regular expressions are used to detect potential PHI such as dates, phone and social security numbers; and (3) word-based dictionary lookups, comparing each word in the document to a database of over 101,000 unique personal and geographic place names built from the U.S. 1990 Census [17].

. *The Medical De-identification System (MeDS)*[7]. MeDS was developed and tested on different types of free-text clinical records such as discharge summaries, clinical notes, and laboratory and pathology reports. This system was especially tuned to process Health Level Seven (HL7) messages [18], but it can easily be modified to accept other document formats. MeDS de-identifies documents through several steps: (1) pre-processing of the well-labeled patient identifiers in report headers (when applicable), and using this information to find the same identifiers in the narrative parts of the report; (2) pattern matching using approximately 40 regular expressions to detect numerical identifiers, dates, addresses, state names and abbreviations, time, ages, e-mail addresses, etc.; (3) name matching with two lists: a list of proper names extracted from different sources (see Table1 for details) and a list of common usage words. Additionally, it searches for predictive markers such as ‘Mr.’ or ‘Dr.’ and uses part-of-speech information to assist with disambiguation of proper names. And, (4) MeDS uses name nearness matching based on a text string nearness algorithm to deal with typographical errors and variants of the patient’s known first, middle and last names.

. *The MIT deid software package*[9]. This software, freely available at the PhysioNet website, was designed to be applicable to a variety of free-text medical records, removing both HIPAA identifiers and an extended PHI set that includes years of dates. The de-identification procedure performs lexical matching with lookup tables, regular expressions, and simple heuristics for context checks. Four types of dictionaries are used by the MIT deid system: (1) lists of known names of patients and hospital staff; (2) lists of generic female and male first names, last names, hospital names, locations and states, all classified as ambiguous or unambiguous items depending on whether they are also common words or not; (3) lists of keywords or phrases that often precede or follow PHI terms; and (4) lists of non-PHI terms such as common words and UMLS terms useful for determining ambiguous PHI terms. Then, PHI instances that involve numerical patterns are identified by regular expressions and context checks. For non-numeric PHI terms, the algorithm first performs dictionary lookups in order to locate known and potential PHI, and then processes several regular expressions that look for patterns with context keywords indicating PHI terms.

## Main characteristics of selected machine learning-based de-identification systems

**Figure 1:**
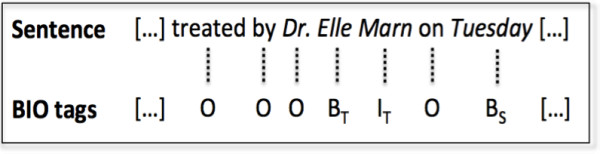
Example of BIO annotations.

. *The MITRE Identification Scrubber Toolkit (MIST)*[10]. MIST provides an environment to support rapid tailoring of automated de-identification to different types of documents. It allows end users to annotate training data and run subsequent experiments. To detect PHI identifiers, MIST uses a machine learning classifier (Conditional Random Fields). and tackles de-identification as a sequence-labeling problem assigning labels to individual words, indicating whether the word is part of a specific type of PHI, or whether it does not belong to the PHI phrase. A well-known encoding using this labeling is the BIO schema; Figure1 depicts an example of BIO annotations. In the figure, B_*T*_ indicates that the word is the beginning of a PHI phrase of type *T*, I_*T*_ indicates a word inside or at the end of a PHI phrase of type *T* and O indicates a word outside a PHI phrase. MIST uses the BIO schema, and is structured to allow the addition of new learning features for the CRF predictions. The default feature specification distributed with MIST only includes a few features; such as the target word, prefixes and suffixes from length 1 to 3, capitalization of the target word and the following word, digits inside the target word, context words denoting a company in the next four words (e.g., “Ltd.”, “Corp.”, “Co.”, “Inc.”), a context window of 3 words, and 2- and 3-grams of words surrounding the target word.

. *The Health Information DE-identification (HIDE) system*[12]. HIDE also provides an environment for tagging, classifying and retagging, which allows the construction of large training datasets without intensive human intervention. For de-identifying documents, HIDE also uses a CRF model for predicting PHI and deals with the problem by tagging each token using the aforementioned BIO schema. The main difference with MIST is that HIDE provides a larger set of features by default. Approximately, 34 features are derived from the morphology of the token (e.g., capitalization, special characters, affixes from length 1 to 3, single- double- triple- and quadruple-digit word, and digits inside, to name but a few); moreover, the context-window processed by HIDE comprises the four previous and four following tokens.

Table1 illustrates a comprehensive summary of the main characteristics of these systems, allowing the reader to have an easy comparison of the systems’ similarities and differences. This information was obtained from the systems’ documentation, as well as from the analysis of their source code files.

**Table 1:** Main characteristics of the de-identification tools

		**HMS Scrubber**	**MeDS**	**MIT deid**	**MIST**	**HIDE**
Main technique	Rule-based	X	X	X	n/a	n/a
	ML-based	n/a	n/a	n/a	X	X
Programming language		Java	Java	Perl	Python	Python
ML algorithm		n/a	n/a	n/a	CRF (Carafe)	CRF (CRFsuite)
Input documents		XML/txt	HL7/txt	txt	txt/XML-inline/json	XML/txt/HL7
HIPAA compliant		X	X	X	^1^	^1^
Regular Expressions (#)		~50	~40	~90	^2^	^2^
PHI markers (e.g., Mr.)		X	X	X	^3^	--
Part-of-speech information		--	X	--	^--^	--
String similarity techniques (e.g. edit distance, fuzzy matching)		--	X	--	--	--
Dictionaries* (size)	Person names	~101K	~280K	~96K^4^	--	--
	Geographic places		~167K	~4K	--	--
	US area code	--	--	~380	--	--
	Medical phrases	--	~50	~28	--	--
	Medical terms	--	~80K	~175K	--	--
	Companies	--	~200	~500	--	--
	Ethnicities	--	~120	~195	--	--
	Common words	--	~220K	~50K	--	--
Machine Learning features	Contextual window	n/a	n/a	n/a	3-words	4-words
	Morphological (#)	n/a	n/a	n/a	22	34
	Syntactic	n/a	n/a	n/a	--	--
	Semantic	n/a	n/a	n/a	--	--
	From dictionaries	n/a	n/a	n/a	^5^	^5^

*HMS Scrubber’s dictionary sources: 1990 US Census (person and place names).

*MeDS’ dictionary sources: Ispell, 2005 SS Death Index, Regenstrief Medical Record System, UMLS, MESH.

*MIT deid’s dictionary sources: 1990 US Census, MIMIC II Database, Atkinson’s Spell Checking Oriented Word Lists, UMLS.

^1^ It will depend on the types of the PHI instances used for training.

^2^ Both MIST and HIDE use regular expression in order to derive the morphological features from the tokens (e.g., all_caps_token ‘^[A-Z] + $’).

^3^ Within MIST, PHI markers are used only for detecting companies (e.g., “Ltd.”).

^4^ Person names dictionaries comprise lists of names, last names and name prefixes.

^5^ These systems are tailored to derive features from dictionaries, however they are not distributed with any default dictionary.

## Evaluation methodology

In order to assess the performance of the selected de-identification tools with VHA clinical documents, we measured the capability of redacting PHI in terms of recall (equivalent to sensitivity here), precision (equivalent to positive predictive value), and F-measure (harmonic mean of recall and precision). Since recall is of paramount importance for de-identification (PHI cannot be disclosed at any rate), we used the F_2_-measure, which weighs recall (twice) higher than precision and is calculated as follows:

(1) $$F_{\beta }-measure=\frac{\left(\beta^{2}+1\right)\cdot precision\cdot recall}{\beta^{2}\cdot precision+recalll}\text{;}\;\beta =2$$

Recall and precision were computed with counts of true positives (system output matches the reference standard), false positives (spurious system output), and false negatives (missed by the system), and we carried out different ways of comparing the system outputs with our reference standard.

## Reference standard corpora

Two different reference standard corpora were used for this study: 1) the *2006 i2b2 de-identification challenge* corpus [19], and 2) a corpus of various VHA clinical documents developed for the CHIR de-identification project.

The i2b2 de-identification corpus includes 889 documents (discharge summaries) that were de-identified and all PHI “re-synthesized” (i.e., automatically replaced with realistic surrogates; further details about the re-synthesis strategy can be found in [19]). This corpus was annotated for 8 categories of PHI and includes 19,498 PHI annotations (details in Table2):

. Patients: includes the first and last name of patients, their health proxies, and family members, excluding titles (e.g., Mrs. *Smith* was admitted).

. Doctors: refers to medical doctors and other practitioners mentioned in the records, excluding titles.

. Hospitals: names of medical organizations and of nursing homes, including room numbers, buildings and floors (e.g., Patient was transferred to *room 900*).

. Locations: includes geographic locations such as cities, states, street names, zip codes, building names, and numbers.

. Dates: includes all elements of a date. Originally, years were not annotated in this corpus, however we modified these annotations in order to consider years and then be consistent with our VHA date annotations.

. Phone numbers: includes telephone, pager, and fax numbers.

. Ages: ages above 90 years old.

. IDs: refers to any combination of numbers, letters, and special characters identifying medical records, patients, doctors, or hospitals (e.g., medical record number).

**Table 2:** PHI category distribution and mapping for the VHA, i2b2 and Swedish Stockholm EPR corpora

**VHA corpus**	**Instances**	**i2b2 corpus**	**Instances**	**Stockholm EPR De-identified Corpus**		**Instances**
Patient Name	206 (3.88%)	Patients	929 (4.76%)	Person Name	First Name	923 (20.87%)
Relative Name	30 (0.55%)					
Other Person Name	20 (0.37%)				Last Name	929 (21%)
Healthcare Provider Name	492 (9.08%)	Doctors	3751 (19.24%)			
Street City	137 (2.53%)	Locations	263 (1.35%)	Location		148 (3.35%)
State Country	161 (2.97%)					
Zip code	4 (0.07%)					
Deployment	43 (0.79%)	-	-	-		-
Healthcare Unit Name	1453 (26.83%)	Hospitals	2400 (12.31%)	Health_Care_Unit		1021 (23.08%)
Other Organization	86 (1.59%)	-	-	-		-
Date	2547 (47.03%)	Dates	7098 (36.40%)	Date_Part		710 (16.05%)
				Full_Date		500 (11.30%)
Age > 89	4 (0.07%)	Ages	16 (0.08%)	Age		56 (1.27%)
Phone Number	90 (1.66%)	Phone Numbers	232 (1.19%)	Phone Number		136 (3.07%)
Electronic Address	4 (0.07%)	-	-	-		-
SSN	16 (0.30%)	IDs	4809 (24.66%)	-		-
Other ID Number	123 (2.27%)			-		-

**Figure 2:**
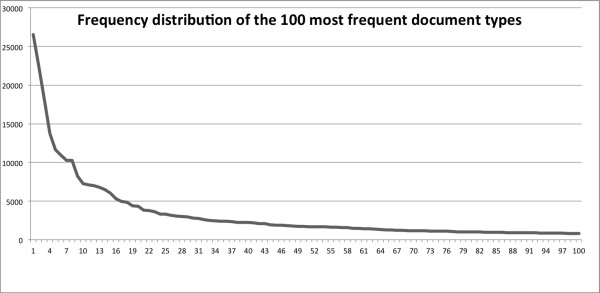
Frequency distribution of the 100 most frequent VHA document types.

The VHA de-identification corpus includes a large variety of clinical documents. A stratified random sampling approach was used to ensure good representation of the variety of clinical notes found at the VHA and a sufficient representation of less common note types. Documents created between 04/01/2008 and 3/31/2009 and containing more than 500 words (to ensure sufficient textual content and PHI identifiers) were included. We then used the 100 most frequent types of clinical notes at the VHA as strata for sampling, and randomly selected 8 documents in each stratum, therefore ending with 800 documents in this corpus. Figure2 depicts the frequency of the 100 most frequent clinical note types (Addendum excluded). These most frequent note types include consult notes from different specialties, nursing notes, discharge summaries, ER notes, progress notes, preventive health notes, surgical pathology reports, psychiatry notes, history and physical notes, informed consent, operation reports, and other less common note types. A few document types correspond to the majority of the clinical notes available. In our case, the 10 most frequent note types correspond to 42% of all clinical notes, and the 25 most frequent note types to 65%.

As already mentioned, an objective of our sampling strategy was to create a reference corpus as much representative of the variety of VHA clinical notes as possible, and although this strategy resulted in oversampling low frequency notes, it also allowed us to measure the generalizability of de-identification tools across document types to a certain extent.

We then manually annotated the documents using a PHI schema that included all categories of PHI defined in the HIPAA “Safe Harbor” legislation [2], as well as some armed forces-specific information such as deployment locations, and units. Each document was independently annotated by two reviewers, disagreements were adjudicated by a third reviewer, and a fourth reviewer eventually examined ambiguous and difficult adjudicated cases. The PHI categories annotated in our corpus were defined as follows:

. Names: all occurrences of person names, distributed in four sub-categories (i.e., patients, relatives, healthcare providers, and other persons) and including first names, last names, middle names and initials (not titles), e.g. “Patient met Dr. *JAMISON JAMES*”.

. Street City: addresses including the city, street number and name, apartment number, etc. (e.g., “lived on *5 Main Street, Suite 200, Albany* NY 0000”).

. State Country: all mentions of states and countries. It also includes mentions of countries associated with military service, service awards, or place of residence at the time of deployment (e.g., “He was awarded the *Korean* service medal”).

. ZIP code: zip code information.

. Deployment: armed forces-specific identifiers that describe a deployment location, or mention of units, battalion, regiment, brigade, etc. (e.g., “had worked as a cook at *Air Base 42* for 3 yrs”).

. Healthcare Unit Name: any facility performing health care services, including smaller units (e.g., detox clinics, HIV clinics), and generic locations such as MICU, SICU, ICU, ER. This also includes all explicit mentions of healthcare facilities, clinical laboratories, assisted living, nursing homes, and generic mentions such as “the hospital”, “the clinic”, or “medical service” (e.g., “patient was referred to the *blue clinic*”, “transferred to *4 west*”).

. Other Organization Name: company or organization names not related with healthcare that are attributed to a patient or provider (e.g., “patient is an active member of the *Elk’s club*”).

. Date: all elements of a date, including year and time, days of the week, and day abbreviations (e.g., “on *December, 11, 2009@11:45am*”, “administered every *Monday*, *TU*, and *Thurs*”).

. Age > 89: all instances of age greater than 89 years old.

. Phone Number: all numeric or alphanumeric combinations of phone, fax, or pager numbers, including phone number extensions (e.g., “call *000-LEAD*”, “dial *x8900*”).

. Electronic Address: references to electronic mail addresses, web pages and IP addresses.

. SSN: combinations of numbers and characters representing a social security number, including first initial of last name and last four digits of the SSN (e.g., “*L0000* was seen in clinic”).

. Other ID Number: all other combinations of numbers and letters that could represent a medical record number, lab test number, or other patient or provider identifier such as driver’s license number (e.g., “prescription number: *0234569*”, “Job *13579/JSS*”).

For the study presented here, a subset of 225 clinical documents was randomly selected from the annotated VHA de-identification corpus. The 225-document evaluation corpus contained 5,416 PHI annotations. Since our objective in this study was to measure how available text de-identification methods perform with VHA documents, to then develop a best-of-breed system adapted for VHA clinical narratives, we had to perform a detailed errors analysis. We decided to use this subset of 225 documents for this study and set part of the corpus aside for future independent evaluation.

In Table2, we show the distribution of each PHI category in our 225-document VHA corpus, along with the corresponding categories in the i2b2 de-identification corpus, as well as the Swedish Stockholm EPR De-identified corpus [20]. Although we did not use the latter in this study, we included it in this table to have another comparison of the distribution of PHI in clinical documents. As shown in the table, Dates, Healthcare Units, and Person Names are the most common PHI categories, while other categories like Ages rarely appear in clinical documents. The PHI category distribution varies significantly between the three different corpora. For example, Locations represent 5.57% of the annotations in the VHA corpus (considering *Street CityState Country* and *Zip code* as Locations), while this percentage drops to 1.35% in the i2b2 corpus and 3.35% in the Swedish Stockholm EPR De-identified corpus. These differences contribute to the difficulties encountered when developing automated text de-identification tools that could be applied across different institutions and document types, a challenge that is still unmet.

## Systems implementation and testing

We carried out an “out-of-the-box” evaluation of each text de-identification system, using their default configuration. Rule-based systems (HMS Scrubber, MeDS, and MIT deid system) used the original regular expression sets, dictionaries and de-identification algorithms implemented by their developers, and were directly tested with our 225 documents reference standard. The machine learning-based systems (MIST and HIDE) had to be trained with annotated samples of text, and were run with their default configuration. We opted for two different methods to train and test these systems: 1) Using the *i2b2 de-identification challenge corpus*[19] for training, and our 225-document corpus for testing. This method used publicly available resources for training and gave us useful information about the generalizability of such systems to different types of corpora. 2) Using our 225-document corpus for training and testing with a *10-fold cross-validation* approach. For this method, the 225-document corpus was randomly split into 10 subsets (or folds) without specific consideration of the document types strata. We then performed the analysis of each fold as the testing data, using the remaining folds for training, and finally averaging the results achieved for each fold to produce a single measurement.

## Systems output analysis

Once the text de-identification tools were run on the evaluation corpus, their output was compared with our reference standard using exact, partial, and “fully-contained” matches, and considering each PHI category separately, or as one ‘PHI’ category.

**Figure 3:**
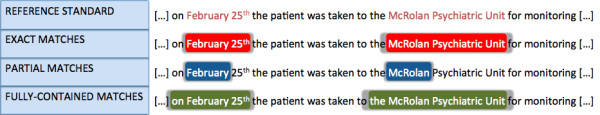
Example of exact, partial and fully-contained matches.s

The different kinds of matches were evaluated as follows (Figure3):

i. *Exact matches* mean that the boundaries or character offset of the predicted PHI have to correspond exactly to the PHI annotation in the reference standard to be considered a true positive.

ii. *Partial matches* consider as correct predictions those with some overlap with the reference PHI annotations. A partial match means that if the prediction partly overlaps with the reference PHI (even only one character), it will be tagged as true positive. This definition of partial matches provides us with a hypothetical performance of the systems if we adapted its redaction techniques to entirely cover our PHI formats.

iii. *Fully-contained matches* try to relax the previous exact matching strategy but ensure all PHI is detected. A fully-contained match therefore considers the prediction as a true positive when it at least overlaps with the entire PHI annotation in the reference standard.

When evaluating system outputs with reference standard matches, all PHI predictions made by a system can be considered as one general PHI category, and then compared with the corresponding unique PHI category in the reference corpus, or each PHI category can be compared separately:

i. *Results with one unique ‘PHI’ category* imply disregarding the specific PHI type of the annotations. Then, when processing the different kinds of matches (i.e. exact, partial or fully-contained) the system annotations are compared with all PHI annotations from the reference.

ii. *Results for each type of PHI separately* involve mapping the PHI categories annotated by each de-identification tool with the different PHI categories specified in our reference standard. To achieve this, we created a series of mappings for each tool we evaluated. Although most tools share many PHI categories with our reference standard (e.g., *Dates*, *Phone Numbers*, *SSN*), other tools’ annotations are less specific, mapping to several PHI categories in our reference standard, or sometimes more specific. For instance, *Names* annotations made by HMS Scrubber could refer to *Patient*, *Relative*, *Healthcare Provider* or *Other Person Names*, and the *Accession Code* and *Medical Record* annotations produced by the MIT deid system both would map to *Other ID Numbers* in our reference standard.

This study has been performed with the approval of the appropriate ethics committee, University of Utah IRB approval reference number IRB_00031374, and the VA Office of Research and Development (approval number 2011-06-007D).

## Results

Tables 3 and 4 summarize the results achieved in the “out-of-the-box” evaluation, grouping them into principally rule-based or machine learning-based methods. For these results, MIST and HIDE were trained using the i2b2 de-identification corpus as described previously.

**Table 3:** **“Out-of-the-box” overall results for using the VHA evaluation corpus exact, partial and fully-contained matches with one *PHI* category, and with each PHI categories separately**

**RULE-BASED SYSTEMS**										
**Overall results**		**Overall results**			**PARTIAL MATCHES**			**FULLY-CONTAINED MATCHES**		
		**HMS Scrubber**	**MeDS**	**MIT deid**	**HMS Scrubber**	**MeDS**	**MIT deid**	**HMS Scrubber**	**MeDS**	**MIT deid**
One PHI	P (CI)	0.01 (0.005-0.015)	0.10 (0.085-0.115)	0	0.32 (0.31-0.33)	0.45 (0.435-0.465)	**0.81** (0.795-0.825)	0.16 (0.15-0.17)	0.14 (0.125-0.155)	0.42 (0.40-0.44)
	R (CI)	0.02 (0.015-0.025)	0.21 (0.20-0.22)	0	0.65 (0.64-0.66)	**0.78** (0.765-0.795)	0.64 (0.625-0.655)	0.34 (0.325-0.355)	0.32 (0.305-0.335)	0.36 (0.345-0.375)
	F_2_ (CI)	0.02 (0.012-0.025)	0.17 (0.16-0.18)	0	0.54 (0.53-0.55)	0.68 (0.665-0.695)	**0.67** (0.655-0.685)	0.28 (0.27-0.29)	0.25 (0.24-0.26)	0.37 (0.355-0.385)
All PHI types	P (CI)	0.01 (0.005-0.015)	0.05 (0.045-0.055)	0	0.23 (0.22-0.24)	0.34 (0.325-0.365)	0.76 (0.745-0.775)	0.12 (0.115-0.125)	0.10 (0.09-0.11)	0.40 (0.335-0.465)
	R (CI)	0.02 (0.0195-0.0215)	0.14 (0.13-0.15)	0	0.47 (0.455-0.485)	0.60 (0.585-0.615)	0.60 (0.585-0.615)	0.26 (0.225-0.295)	0.22 (0.205-0.235)	0.34 (0.325-0.355)
	F_2_ (CI)	0.02 (0.018-0.022)	0.10 (0.09-0.11)	0	0.39 (0.38-0.40)	0.52 (0.505-0.535)	0.63 (0.615-0.645)	0.21 (0.195-0.225)	0.18 (0.17-0.19)	0.35 (0.315-0.385)
**MACHINE LEARNING-BASED SYSTEMS**										
Overall results			**EXACT MATCHES**		**PARTIAL MATCHES**		**FULLY-CONTAINED MATCHES**			
			MIST	HIDE	MIST	HIDE	MIST		HIDE	
One PHI	P (CI)		0.54	0.50	**0.95**	0.89	0.58		0.56	
			(0.52-0.56)	(0.48-0.52)	(0.935-0.965)	(0.875-0.905)	(0.56-0.60)		(0.54-0.58)	
	R (CI)		0.25	0.27	0.46	**0.49**	0.28		0.30	
			(0.24-0.26)	(0.26-0.28)	(0.445-0.475)	(0.475-0.505)	(0.27-29)		(0.29-31)	
	F_2_ (CI)		0.28	0.30	0.51	**0.54**	0.31		0.33	
			(0.265-0.295)	(0.285-0.315)	(0.495-0.525)	(0.525-0.555)	(0.295-0.325)		(0.315-0.345)	
All PHI types	P (CI)		0.52	0.48	0.90	0.84	0.55		0.52	
			(0.495-0.545)	(0.46-0.50)	(0.885-0.915)	(0.825-0.855)	(0.525-0.575)		(0.50-0.54)	
	R (CI)		0.24	0.25	0.44	0.46	0.27		0.28	
			(0.225-255)	(0.24-0.26)	(0.425-0.455)	(0.445-0.475)	(0.255-0.285)		(0.265-0.295)	
	F_2_ (CI)		0.27	0.28	0.49	0.50	0.30		0.31	
			(0.255-0.285)	(0.265-0.295)	(0.475-0.505)	(0.485-0.515)	(0.285-0.315)		(0.295-0.325)	

*CI*: Confidence Interval obtained with a confidence level of 95%.

One PHI = one overall PHI category considered.

All PHI types = each PHI type evaluated separately.

*P* = Precision; *R* = Recall; *F*_*2*_ = F_2_-measure.

**Table 4:** “Out-of-the-box” individual PHI recall results for partial and fully-contained matches using the VHA evaluation corpus

**RULE-BASED SYSTEMS**							
**PHI type**	**#Inst.**	**PARTIAL MATCHES**			**FULLY-CONTAINED MATCHES**		
		**HMS Scrubber**	**MeDS**	**MIT deid**	**HMS Scrubber**	**MeDS**	**MIT deid**
Patient Name	206	0.83	**0.99**	0.98	0.57	0.69	0.69
Relative Name	30	0.76	0.95	1	0.57	0.67	0.77
Healthcare Provider Name	492	0.74	0.96	0.94	0.43	0.47	0.38
Other Person Name	20	0.66	0.81	0.74	0.30	0.25	0.35
Street City	137	0.90	0.96	0.81	0.70	0.78	0.78
State Country	161	0.45	0.49	0.85	0.43	0.45	0.84
Deployment	43	0.34	0.49	0.27	0.07	0.02	0.05
ZIP code	4	1	1	1	1	1	1
Healthcare Unit Name	1453	0.45	0.51	0.12	0.24	0.23	0.03
Other Org Name	86	0.33	0.50	0.27	0.03	0.20	0.03
Date	2547	0.74	0.87	0.80	0.34	0.27	0.46
Age > 89	4	0	0	1	0	0	1
Phone Number	90	0.73	0.79	0.80	0.42	0.5	0.48
Electronic Address	4	0	0.86	0.75	0	0	0.75
SSN	16	1	1	1	1	1	1
Other ID Number	123	0.66	0.82	0.41	0.43	0.61	0.27
**MACHINE LEARNING-BASED SYSTEMS**							
PHI type	**#Inst.**	**PARTIAL MATCHES**			**FULLY-CONTAINED MATCHES**		
		MIST	HIDE		MIST	HIDE	
Patient Name	206	0.51	0.54		0.42	0.50	
Relative Name	30	0.13	0.13		0.13	0.13	
Healthcare Provider Name	492	0.53	0.59		0.44	0.53	
Other Person Name	20	0	0.20		0	0.15	
Street City	137	0.26	0.29		0.26	0.27	
State Country	161	0.14	0.22		0.14	0.21	
Deployment	43	0.07	0.05		0.07	0.02	
ZIP code	4	0	0.75		0	0.75	
Healthcare Unit Name	1453	0.09	0.09		0.06	0.05	
Other Org Name	86	0.09	0.07		0.06	0.06	
Date	2547	0.72	0.73		0.39	0.38	
Age > 89	4	0	0		0	0	
Phone Number	90	0.34	0.61		0.24	0.51	
Electronic Address	4	0	0		0	0	
SSN	16	0.56	0.87		0.56	0.87	
Other ID Number	123	0.32	0.69		0.20	0.63	

Table3 shows the overall results in terms of precision, recall and F_2_-measure, with one unique *PHI* category, and with all PHI types, for exact, partial and fully-contained matches. Confidence intervals (CI) were computed using a confidence level of 95%.

Table4 presents recall (i.e., sensitivity) values for each PHI type considering partial and fully-contained matches. In this table, exact match results were not reported because the systems were not designed following our annotation guidelines and often add word delimiters or other characters to their annotations that don’t correspond to the exact annotations we have in our reference standard. Therefore, we believe that partial and fully-contained matches are more relevant in this study and give us more useful information about the suitability of these systems to de-identify VHA clinical documents. Also, Table4 only presents recall measurements because of our emphasis on PHI detection and removal rather than correct PHI type assignment, and because the mappings between the predicted PHI and the reference standard are often not one-to-one mappings and prevented us from determining the PHI type source of false positives, and consequently the individual PHI type precision values.

Tables 5 and 6 show, in the same fashion as the previous two tables, the results of the 10-fold cross-validation experiment carried out with the machine learning-based de-identification tools (MIST and HIDE). Table5 presents the overall precision, recall and F_2_-measure results for the three kinds of matches with both one unique *PHI* category and each individual PHI types. Table6 provides recall values for each PHI type.

**Table 5:** **10 fold cross-validation overall results using the VHA evaluation corpus for exact, partial and fully-contained matches with one *PHI* category, and with each PHI types separately**

**10-fold cross-validation experiment**							
**Overall results**		**EXACT MATCHES**		**PARTIAL MATCHES**		**FULLY-CONTAINED MATCHES**	
		**MIST**	**HIDE**	**MIST**	**HIDE**	**MIST**	**HIDE**
One PHI	P (CI)	0.89	0.88	**0.96**	0.95	0.91	0.91
		(0.88-0.90)	(0.87-0.89)	(0.95-0.97)	(0.94-0.96)	(0.90-0.92)	(0.90-0.92)
	R (CI)	0.64	0.70	0.70	**0.76**	0.67	0.73
		(0.625-0.655)	(0.685-0.715)	(0.685-0.715)	(0.75-0.77)	(0.655-0.685)	(0.72-0.74)
	F_2_ (CI)	0.68	0.73	0.74	**0.79**	0.71	0.76
		(0.665-0.695)	(0.72-0.74)	(0.725-0.755)	(0.775-0.805)	(0.70-0.72)	(0.75-0.77)
All PHI types	P (CI)	0.87	0.87	0.95	0.92	0.90	0.89
		(0.855-0.885)	(0.86-0.88)	(0.94-0.96)	(0.905-0.935)	(0.885-0.915)	(0.88-0.90)
	R (CI)	0.63	0.69	0.69	0.74	0.66	0.71
		(0.615-0.655)	(0.675-0.705)	(0.675-0.705)	(0.725-0.755)	(0.645-0.675)	(0.695-0.725)
	F_2_ (CI)	0.67	0.72	0.73	0.77	0.70	0.74
		(0.655-0.685)	(0.71-0.73)	(0.713-0.745)	(0.76-0.78)	(0.685-0.715)	(0.725-0.755)

*CI*: Confidence Interval obtained with a confidence level of 95%.

One PHI = one overall PHI category considered.

All PHI types = each PHI type evaluated separately.

*P* = Precision; *R* = Recall; *F*_*2*_ = F_2_-measure.

**Table 6:** 10 fold cross-validation recall results for partial and fully-contained matches by PHI type and using the VHA evaluation corpus

**10-fold cross-validation experiment**					
**PHI type**	**#Inst.**	**PARTIAL MATCHES**		**FULLY-CONTAINED MATCHES**	
		**MIST**	**HIDE**	**MIST**	**HIDE**
Patient Name	206	0.51	0.51	0.49	0.51
Relative Name	30	0	0.13	0	0.10
Healthcare Provider Name	492	0.58	0.61	0.54	0.59
Other Person Name	20	0	0.20	0	0.20
Street City	137	0.28	0.48	0.28	0.43
State Country	161	0.58	0.71	0.58	0.70
Deployment	43	0.19	0.28	0.16	0.21
ZIP code	4	0	0	0	0
Healthcare Unit Name	1453	**0.55**	**0.61**	**0.52**	**0.58**
Other Org Name	86	0.10	0.29	0.09	0.25
Date	2547	**0.93**	**0.94**	**0.89**	**0.92**
Age > 89	4	0	0	0	0
Phone Number	90	0.27	0.88	0.23	0.78
Electronic Address	4	0.75	0.75	0	0.75
SSN	16	0.37	0.62	0.37	0.56
Other ID Number	123	0.37	0.72	0.34	0.65

## Discussion

### “Out-of-the-box” evaluation

Results of this first evaluation (Table3) demonstrate the difficulties rule-based systems have to determine the exact boundaries of our PHI annotations. We expected such low results since the regular expressions and de-identification algorithms implemented in these systems were designed with different PHI annotation guidelines, different clinical documents, and in different institutions. As mentioned earlier, they often add word delimiters to the PHI annotations, or some text portions that are not part of the reference annotations (e.g., ‘JOB#’ in “JOB#000201” or ‘Dr.’ in “Dr. Sands”). Machine learning-based systems obtain better exact match results, suggesting that some resources, algorithms, and features like the ‘BIO’ schema used by these systems to determine PHI boundaries could be suitable for de-identifying our VHA documents.

As expected, Table3 illustrates that the best results are achieved with partial matches and one unique *PHI* category (95% precision, 78% recall and 67% F_2_-measure), decreasing dramatically when considering fully-contained matches (58% precision, 36% recall, 37% F_2_-measure). It indicates the need for adapting the de-identification methods and resources to our PHI specifications and documents. However, partial matches results are quite encouraging, suggesting that, after adaptation, we could reach at least such performance.

In general, results with one unique *PHI* category (i.e., without considering mapping between PHI types) are better than with each PHI type analyzed individually, especially for rule-based systems. To our understanding, there are two reasons for this: 1) the differences between some PHI types are quite small, and regular expressions used to detect a specific PHI type also recognize other PHI types (e.g., *Phone Numbers* and *Other ID Numbers*); and 2) sometimes dictionary entries are common to more than one PHI type, causing ambiguity issues (e.g., ‘Aurora’ could be a city, person name, or the name of a hospital). In contrast, for machine learning-based systems the differences with one unique *PHI* category and all PHI types are less significant. We believe that this is because the training features used by the systems are mainly based on the context (i.e., surrounding words and characters) and morphology of the words, and don’t rely on fixed patterns or dictionary entries. This may cause these systems to achieve lower recall but seems to make them more precise.

In general, rule-based systems achieve better recall values overall, but lower precision than machine learning-based systems. However, it is worth mentioning that the MIT deid system obtained a precision of 81% with partial matches, which points out that its pattern matching techniques and dictionaries will be useful to accurately de-identify our documents. For the machine learning-based systems, precision was always higher, but recall usually lower. This is not surprising since the training models were created using the i2b2 corpora, which does not correspond enough to our clinical documents. Furthermore, the lack of learning features derived from dictionaries has also an impact on recall issues.

For de-identification, recall (i.e., sensitivity) is the most important measure to take into account. Personal identifiable data cannot be revealed under any circumstance, it is therefore more important to get high sensitivity when obscuring PHI than higher precision. Table4 shows the recall values for each individual PHI type. As with overall results, rule-based systems achieved better recall, especially with some PHI types such as *Zip codes*, *Age > 89* and *SSN*. Techniques based on pattern matching clearly seem to be the most appropriate for these types of PHI.

Names in general, and especially *Patient Names*, are also well de-identified by rule-based systems, reaching the highest (partial match) recall of 99% by MeDS. Location information (*Street City*, *State Country*) and *Electronic Address* are also properly covered by at least one rule-based system. This fact suggests that dictionaries of names, cities and countries, as well as the patterns used by these systems for names, cities, countries, postal addresses, e-mails and webpages are useful for our purposes; and they will be considered for future implementations.

These results also show that some of our PHI types need new methods or strategies for de-identification. For instance, the recall obtained for *Deployment*, *Healthcare Unit Name* and *Other Organization Name* is somewhat low and needs improvements. This is probably due to: 1) *Deployment* annotations being almost exclusive to characteristics of VHA documents, and not specified in the HIPAA legislation; 2) *Healthcare Unit Name* identifiers involving numerous acronyms and abbreviations that were often missed (e.g., OT (Occupational Therapy), ER (Emergency Room)); and 3) *Other Organization Name* being a very ‘open’ category (even if some systems included dictionaries of company names).

Machine learning-based systems are more precise when determining PHI boundaries, and interestingly performed well with several PHI types like *Dates*, *Phone Numbers* and *Other ID Numbers* that supposedly should be properly detected by pattern matching techniques. We will report reasons for that when analyzing systems predictions.

### Ten-fold cross-validation experiment

As previously mentioned, this experiment was the first adaptation of machine learning-based de-identification tools to VHA clinical documents. The main objective of this experiment was to analyze the performance of two machine learning-based text de-identification tools with their default configuration and with training and testing using our VHA documents.

Table5 presents the overall results for this experiment, which –as expected– significantly surpass the ones obtained by these systems when trained with the *i2b2 de-identification corpus*. For the three types of matches, precision was good, with values between 87% and 96%; and recall was higher (63%-76%), allowing for a higher 79% F_2_-measure.

These results demonstrate again that machine learning-based tools allow for better precision than rule-based systems, although a need for recall improvement is still present.

When examining performance with each individual PHI type (Table6), recall was relatively low with several PHI types such as *Relative Name*, *Other Person Name*, *Deployment*, *Zip code*, *Other Organization Name* and *Age > 89*. This low performance was due to the lack of features tailored to specific PHI types, as well as to the scarcity of training examples in a few PHI types (e.g., only 4 examples of *Zip code* and *Age > 89* in our evaluation corpus). However, when the number of training examples increases, the impact of specific training features lessens, as exemplified with *Healthcare Unit Names* and *Dates* (more than 2,500 training examples in our evaluation corpus).

Among machine learning-based systems, one was clearly better when redacting numerical PHI identifiers such as *Phone Number* and *Other ID Number*. We believe that this difference occurs because this system uses several numerical learning features such as *is-a-4digits-token* and *is-a-CharsNumbers-token*, which are not found in the default feature specification of the other system, and allow for more discriminating training models for these PHI types. This demonstrates the importance of learning features adapted to the target PHI type.

### Partial matches analysis

**Figure 4:**
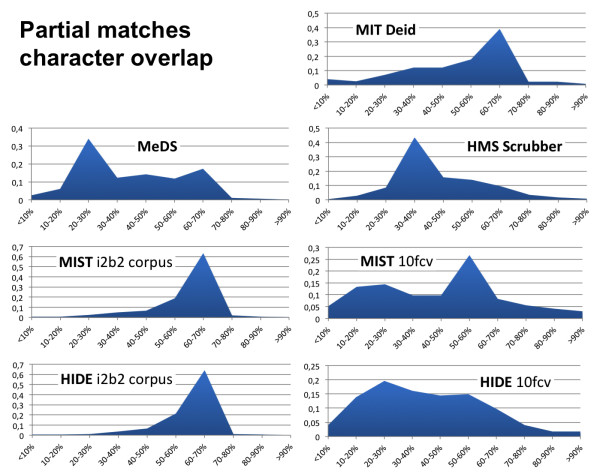
Partial matches character overlap analysis.

To have a more precise insight of partial matches, we analyzed the percentage of overlapping characters of all partial matches. As shown in Figure4, most systems had partial matches with between 20% and 70% characters overlap. Rule-based systems had partial matches that mostly fell into the 20-40% character-overlapping rate, which means that considerable adaptations of their algorithms would be needed. In contrast, the MIT deid system had partial detections overlapping between 50 and 70%, indicating that its algorithms work better with our PHI formats. Finally, machine learning-based systems trained using the i2b2 corpus made partial predictions that mostly overlapped between 60 and 70%, while for the 10-fold cross-validation experiment the predominant category was 20-30% for HIDE and 50-60% for MIST. Therefore, although the overall recall for MIST and HIDE trained with the i2b2 corpus was quite low (Table3), the partial detections they produced were somewhat accurate in terms of characters overlap. The lower overlap rate reached with the 10-fold cross-validation experiment demonstrates a need for more training examples and advanced learning features that make partial detections more accurate.

### Systems errors analysis

Besides measuring the performance of the de-identification systems, we also performed a detailed errors analysis. The main observations of this analysis are detailed below.

Main causes of missing PHI annotations (i.e., false negatives):

. *Unusual PHI formats*: Some PHI types appear within our documents in unusual formats that are not always detected by the de-identification systems. Rule-based systems missed these annotations because none of their regular expressions capture such formats; while for machine learning-based systems, these annotations were missed because of differences between the training and testing feature vectors or because of a lack of useful training features capturing these atypical PHI formats. However, the de-identification systems sometimes partially captured these unusual formats, as reflected by an increase in partial matches. For example, the instance of *Dates* ‘Jan 09, 2000@07:54:32’ was partially detected by all three rule-based de-identification systems, and missed by the machine learning-based systems when trained with the i2b2 de-identification corpus. Other examples of missing annotations were phone numbers that included country codes, and day intervals such as ‘WFS’ or ‘M-F’.

. *Acronyms and abbreviations*: The *Healthcare Unit Names* PHI type includes many acronyms and abbreviations referring to healthcare facilities that are frequently missed by all five de-identification systems. For instance, ‘MH’ (Mental Health), ‘ECF’ (Extended Care Facility), or ‘ENT’ (Ear, Nose, Throat) were always missed by all systems. A possible reason for this is that these systems were developed with a different definition of what a PHI is. Concluding that a term is PHI or not is at times a judgment call. Admittedly, in our evaluation we defined terms that are PHI in a relatively broad sense. It is conceivable that others may be of the opinion that these terms do not meet the strict definition of PHI.

. *Lack of examples*: This mainly affects machine learning-based systems; and it causes PHI patterns with few instances in the corpus to be missed. For instance, less than 10 instances of *Dates* formats like ‘020309’ or ‘70’s’ were found in our corpus, and they were often missed. This of course highlights one of the key weaknesses of all machine learning based systems: lack of training data.

Main causes of spurious PHI annotations (i.e., false positives):

. *Measurements*: A common error committed by all systems consisted in confusing medical measurements with *Dates* or other numerical identifiers. For instance, in the phrases “rating: 1/10” and “maintain CVD 8-10”, ‘1/10’ and ‘8-10’ were annotated as *Dates*.

. *Ambiguous words*: Common words, which could also represent PHI, were often wrongly annotated by the systems, especially by rule-based de-identification systems. For instance, ‘BROWN, ‘GRAY’, ‘WALKER’ or ‘CUTTER’ are examples of ambiguous words that were sometimes wrongly recognized as PHI by the systems.

. *De-identification systems’ specific PHI types*: A few PHI types annotated by de-identification systems were not included in our PHI specification. For example, HMS Scrubber annotates expressions like ‘30 days’ as ages; however, these annotations are not considered PHI in our reference standard.

This errors analysis emphasizes the need for adaptation of methods used by de-identification systems in order to recognize PHI formats specific to VHA clinical documents. It is also clear that in general, system adaptation is required to achieve accurate de-identification of corpora the system was not developed or trained on. Therefore, it is important to evaluate system adaptation in terms of the time, effort and complexity required. For example, compared to the annotation effort required by machine learning-based systems, do rule-based de-identification systems require less adaption time and effort to achieve acceptable de-identification performance on disparate corpora? This is an area we hope to study in future research. Also, improvements in the analysis of the local context, VHA-specific dictionaries, and lists of trigger words could be beneficial to disambiguate acronyms, abbreviations, as well as VHA-specific PHI types like *Deployment*. Finally, the large number of false positives introduced by rule-based methods indicates the need for more sophisticated techniques to filter them out and make these methods more accurate. The question that remains however is can accurate filtering of false positives be achieved while still maintaining high recall (sensitivity).

### Limitations

Although our VHA corpora include many different types of clinical notes, the statements presented here are based on our observations running the selected de-identification systems on VHA clinical documents, and don’t necessarily generalize to other organizations and clinical document types.

Another limitation of this study is the size of the training corpus used in the 10-fold cross-validation experiment. This limited size could have repercussions on the training phase of machine learning-based systems. However, we believe that although small in size, our evaluation corpus provides useful insights about the overall performance that can be achieved by these systems. And as previously mentioned, it shows that precision is well addressed by machine learning-based systems, and we will use this knowledge for future improvements and developments of de-identification tools.

## Conclusions

We have presented a study about the suitability of current text de-identification methods and tools for de-identifying VHA clinical documents. In this study, five de-identification tools were selected and evaluated with a corpus of VHA clinical documents. The “out-of-the-box” evaluation performed in this study helped us determine how the default configuration of these tools addressed the de-identification of our documents, and more importantly, informed us about the best methods and resources for our “best-of-breed” VHA text de-identification tool. This “out-of-the-box” evaluation showed us that some PHI types such as *SSN* and *Zip code* are clearly recognized better by rule-based methods. Also, when considering partial matches, *Names* and other ‘textual’ PHI are well de-identified by the algorithmic procedures developed by some rule-based systems (MeDS and the MIT deid system). The results achieved with the 10-fold cross-validation experiment show that machine learning-based systems obtained better precision; methods based on machine learning algorithms could therefore play an important role to improve the overall precision of de-identification systems.

These observations convinced us that adapting the techniques used by the rule-based systems to our documents, and using enriched feature sets and training examples for machine learning-based approaches, could lead to satisfactory de-identification of VHA clinical documents. Rule-based inferences would be used to obtain a high sensitivity, and machine learning approaches would be applied to filter out the false positives produced by rule-based methods. Therefore, although this study showed that none of the tested systems reported sufficient “out-of-the-box” performance to de-identify our VHA clinical documents, it gave us compelling insight into the best methods to use when developing our VHA de-identification tool.. This tool will take advantage of the methods used by the five systems tested here, and also implement new techniques when needed. To achieve this, we will adapt the rule-based techniques to the format of our PHI identifiers as well as improve the machine learning models.

Finally, we conclude with two issues that will lead future discussions and reinforce the need for developing accurate automated de-identification systems: 1) the risk of re-identification; and 2) the interpretability of de-identified documents.

Obviously, the better the de-identification, the lower the risk of re-identification. However, beyond de-identification techniques, there is always a potential risk of re-identification. De-identification is not anonymization. Several studies [21] have demonstrated that a combination of specific pieces of information or events (even if they are not considered PHI by the HIPAA “Safe Harbor” method) could provide enough evidence to identify an individual. This fact proves the strong need for accurate de-identification tools, and probably more conservative approaches than the HIPAA “Safe Harbor” technique (e.g., in our VHA corpus we followed the HIPAA guidelines, but also annotated all states, countries, all elements of dates including years, and deployment information, which could uniquely identify a veteran), possibly adapting some anonymization algorithms to narrative text.

On the other hand, depending on the target research study, de-identified narratives may not be useful (e.g., statistics about specific zip codes). Furthermore, if the de-identification tool is extremely oriented toward patient confidentiality protection (e.g., by removing all portions of text that are not considered medical concepts, as experimented by Berman [22]), the interpretability and usefulness of the documents can be severely compromised, making any subsequent analysis like information extraction extremely arduous.

## Competing interests

Most authors (OF, BRS, SS, MHS, SMM) declare that they have no competing interests. FJF is the author of one of the text de-identification tools evaluated, and therefore had only limited involvement.

## Authors’ contributions

OF prepared the de-identification tools, ran them, analyzed their performance, and drafted the manuscript. BRS and SS led the development of the VHA documents reference standard. Both helped draft the manuscript, along with FJF. SMM conceived the evaluation, selected the de-identification tools to evaluate, analyzed their performance, and drafted the manuscript. MHS revised the manuscript critically and in details. As the author of one of the de-identification tools we evaluated, FJF didn’t participate in the preparation and testing of the de-identification tools. All authors read and approved the final manuscript.

## Pre-publication history

The pre-publication history for this paper can be accessed here:

http://www.biomedcentral.com/1471-2288/12/109/prepub
